# Assessing cyclists’ routing preferences by analyzing extensive user setting data from a bike-routing engine

**DOI:** 10.1186/s12544-021-00499-x

**Published:** 2021-07-27

**Authors:** Michael Hardinghaus, Simon Nieland

**Affiliations:** 1 German Aerospace Center DLR, Institute of Transport Research, Rudower Chaussee 7, 12489 Berlin, Germany; 2 Department of Geography, Humboldt University of Berlin, Unter den Linden 6, 10099 Berlin, Germany

**Keywords:** Active travel, Bicycle route choice, Navigation data, Preference types

## Abstract

**Introduction:**

Many municipalities aim to support the uptake of cycling as an environmentally friendly and healthy mode of transport. It is therefore crucial to meet the demand of cyclists when adapting road infrastructure. Previous studies researching cyclists’ route choice behavior deliver valuable insights but are constrained by laboratory conditions, limitations in the number of observations, or the observation period or relay on specific use cases.

**Methods:**

The present study analyzes a dataset of over 450,000 observations of cyclists’ routing settings for the navigation of individual trips in Berlin, Germany. It therefore analyzes query data recorded in the bike-routing engine BBBike and clusters the many different user settings with regard to preferred route characteristics.

**Results and Conclusion:**

Results condense the large number of routing settings into characteristic preference clusters. Compared with earlier findings, the big data approach highlights the significance of short routes, side streets and the importance of high-quality surfaces for routing choices, while cycling on dedicated facilities seems a little less important.

Consequentially, providing separated cycle facilities along main roads – often the main focal point of cycle plans – should be put into the context of an integrated strategy which fulfills distinct preferences to achieve greater success. It is therefore particularly important to provide a cycle network in calm residential streets as well as catering for short, direct cycle routes.

## Introduction

Regarding negative external effects, the bicycle is an attractive mode of transport. In recent years, many western cities have seen an increase in cycling rates [1–3]. Most recently, due to the covid-19 pandemic bicycle use increased strongly while utilization of public transport declined [3–5]. This stresses the long-term importance of supporting the bicycle as alternative mode of transport and as feeder to boost public transport [6]. One important measure aimed at supporting the uptake of cycling is adapting the urban infrastructure to meet the demand of cyclists. Accordingly, cyclists’ route choice behavior and preferences are highly relevant in planning and practice. To assess this behavior, previous studies are based mainly on two research approaches:

- In revealed preference studies (RP), the actual behavior is observed. Most recent studies track cyclists and compare the route chosen to potential alternatives to evaluate the impact of route characteristics on route choice [7–10].
- In stated preference studies (SP), participants take decisions based on a set of hypothetical alternatives. In an interview setting, probands choose between defined route descriptions which normally differ in route characteristics and travel time [11–14].

Apart from these two main research paradigms, studies differ widely from each other when looking at the parameters under observation. Different investigation areas also vary in terms of the local significance of bicycle transport or the network as is. The latter is very important when defining alternatives in revealed preference studies. In general, earlier research found that short travel times and routes avoiding disturbance by motorized transport were more preferred [8, 11, 14]. Studies with different contexts therefore deliver varying findings when it comes to the importance of route characteristics that ensure fewer disturbances. For instance, some studies see calm side streets as a first choice [8, 15] while others conclude that separated facilities are preferred [11, 16]. The importance of smoother pavements or paved over unpaved road is demonstrated [10] but, in the context of other route characteristics, their importance is limited [16]. These prior studies related to two different approaches (RP and SP) enabled to gain a good understanding of the complex route choice behaviour.^1^

Although using well developed and broadly accepted methods, any overt survey situation involves response biases, such as the observer bias [17] or social desirability bias [18], which potentially distort the results. In addition, the observation periods and sample sizes are limited due to extensive and costly data collection. Both paradigms (RP and SP) also have individual strengths and limitations [19, 20]. The hypothetical nature of choice experiments often leads to an overestimation of the willingness to pay [21]. In addition, by the example of recreation research other limitations like the perception or image of the alternatives as well as the estimation of context effects in relation to the range of levels provided are shown [22]. Researching route choice behavior also reaches limits because stated choice sets can only present single route segments. In real-life, a route is normally composed of several varying route segments, including real-life constraints. For instance, a route along side streets is usually more complex than cycling along a main road, which cannot always be captured by standard measures of detour or expenditure of time. On the other hand, revealed preference studies depend heavily on the given network in the observation area. It is not possible to evaluate infrastructure elements that are not present as attractive alternatives to the participants. Likewise, the research may include alternatives that may be unknown to the participants. Either way, the individual choice of a certain route may have other reasons that are not observed. In recent years, big data methods are being applied in cycling research [23, 24]. While these approaches are mainly focused on bike sharing, further promising data sources on every day cycling have the potential complete the picture of route preferences.

This paper uses requests of a bike-routing engine to derive cyclists routing preferences from user settings in the context of bike navigation. This refers to individual settings which are stated by the user to specify the navigation according to the users’ desires for each individual trip regarding various route characteristics. The objective of this study is therefore to deliver insights into the desired routing characteristics of urban cycle journeys following a different approach than classical RP or SP studies. It analyzes cyclists’ recorded settings when performing routing requests in a clustering procedure to derive typical types of routing preferences. They are based on the large variety of possible combinations of user settings as users may specify graduated routing preferences in the input fields *street category*, *surface quality* and *green pathways*. Accordingly, appropriate routes are suggested for the origin-destination relations based on these settings. Based on the data, the number of requests for each preference type is analyzed in order to evaluate its importance. This enables us to derive recommendations for planning and practice.

The research is conducted in Berlin using the bike-routing engine BBBike [25]. This is an appropriate case, as the local bike-routing engine has a long history and high usage level. In addition, standing at 18% in 2018, Berlin has a substantial mode share of cycling [26].

## Methods

Recently, search engine data (mainly Google Trends or internet search queries) is being used for various research questions like estimating future tourism demand [27], modelling suicide rates [28] or evaluating the perception of mental health in the context of mass shooting events [29]. Using these data in the present approach allows us to validate results derived from classical approaches by researching route searching behaviour. The approach has advantages compared to SP or RP studies: first, there are no laboratory conditions or any survey situation when gathering the data. The queries raised by regular users are recorded in the back end. These real-life conditions promise a rather realistic picture since they include given interrelations between different characteristics as well as side-effects, such as a less direct route when intentionally avoiding main roads. Second, the sample size of the analyzed dataset is large. The data collection method enables us to record and analyze a full sample of users of the bike-routing engine with almost half a million queries. Third, the observation period is long. The data is collected over a whole year. Finally, the structure of the data means that we can use a relatively simple analysis method. Compared with rather complex modelling approaches, when looking at stated preference studies or difficulties when generating alternatives in revealed preference studies, the present approach uses simple hierarchical clustering.

The methodological approach of the present study contains five steps. First, data is gathered by recording the request in the bike-routing engine. Second, the data is preprocessed and transformed to a consistent geographical reference system. Third, the data is explored and compared with municipal household survey data. Fourth, a hierarchical clustering is performed to derive preference types. Finally, preference types are described and the importance of each preference type is evaluated.

### Data basis

The main component of the present study is analyzing data recorded by BBBike. BBBike is a bike-routing engine for cyclists. The initial version was developed in 1999 for the city of Berlin [25]. It is now available in many towns and cities. The software is accessible via web browser and as a mobile app. BBBike searches cycle routes between two points. After choosing for origin and destination of the trip, the setting menu opens. Users are encouraged to specify routing preferences with various settings as shown in Table 1. After confirming the settings, the route is calculated.

**Table 1:** Characteristics available for routing requests in the BBBike bike-routing engine (http://www.bbbike.de)

Variable	Value
Speed	Free field, default is 20 km/h
Street category	No preference
	Prefer residential roads [calm]
	Use only residential roads [calm*]
	Prefer main roads [main]
	Use only main roads [main*]
	Avoid main roads without cycle paths/bus lanes [infra]
	Avoid main roads without cycle paths [infra*]
Surface quality	No preference
	Avoid cobblestones and bad surfaces [smooth]
	Use only very good surfaces (suitable for racing bikes) [smooth*]
Avoid traffic lights	No
	Yes
Avoid unlit streets	No
	Yes
Green pathways	No preference
	Prefer green pathways [green]
	Strongly prefer green pathways (may result in longish routes if there are no suitable routes surrounded by greenery available, so use with caution) [green*]
Use unknown streets	Allow routing through “unknown” streets (streets which are not yet researched for cyclist usage)

The proposed route is described, can be displayed on an interactive map and exported in various formats. The interactive map can display cycle paths, surface quality, public transport, greenery and other data and can also show current weather conditions. The bike-routing engine uses the OpenStreetMap (OSM) road network for routing [30]. The street network in the investigation area is diverse. With regard to the total length of the street network, our own calculations based on the OSM describe the infrastructure as follows: 69% is assigned to residential roads and 17% to main roads. Of the main roads, 39% of the length is covered by a cycle infrastructure, while 27.5% of the municipal area is green area. In the side street network, a significant number of streets have cobblestones or bad surfaces. Residential roads almost never have any cycle infrastructure.

**Fig. 1:**
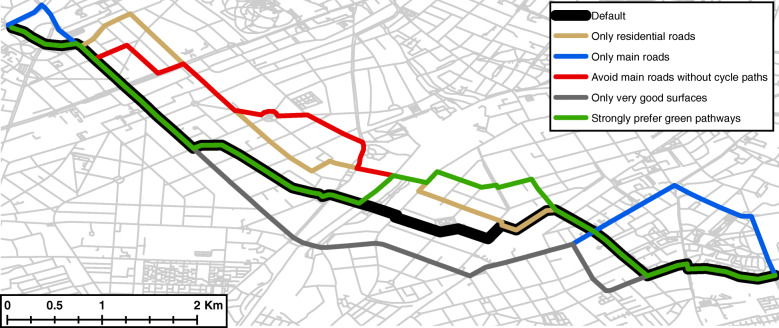
Different routing suggestions for the origin-destination relation between two university locations in Berlin. The routes are between 6573 and 7440 m long; the overlap with the default route ranges from 20 to 76%

Based on the differences in the road network and the level of detail the routing engine provides, the suggested routes vary widely from each other depending on the preference settings. Based on the routing preference settings, the routes for the same origin-destination relation can be up to one third longer compared with the shortest route (the default setting). Accordingly, routes under different settings may overlap completely or not have any segment in common (see Fig. 1). Figure 1 shows different routes for the origin-destination relation between two university locations in Berlin. These routes are between 6573 and 7440 m long; the overlap with the shortest route under default settings ranges from 20 to 76%. For a detailed overview of the length and overlap of different routes for varying settings, see Table 2 in appendix and figures in the appendix. These provide an impression of the sensitivity of the routing algorithm. These routes display one specified routing preference per alternative. Since the routing preferences in the individual categories can be combined the resulting variety of proposed routes in very large.

For this study, all requests in the city of Berlin were logged over a period of 1 year (the whole of 2017), including the timestamp of the request, start and end point coordinates, addresses and postcodes as well as all user settings regarding the routing preferences for the individual trip. In total, the observation period covers 461,170 valid requests, an average of approximately 1263 per day.

**Fig. 2:**
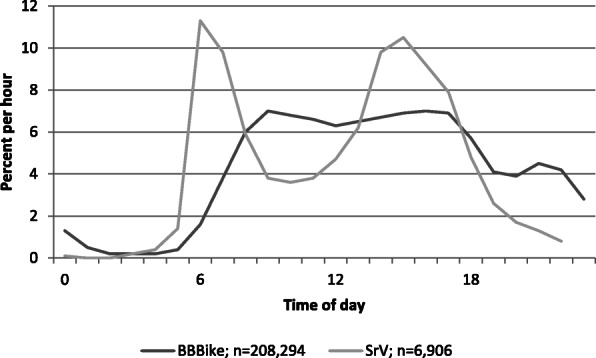
Distribution of BBBike requests over the course of the day compared with the municipal survey. Both curves show the average for weekdays Tuesday to Thursday

Due to methodological reasons, the sample does not show representative data for all trips travelled by bicycle. As shown in Fig. 2, compared with the municipal household travel survey data (SrV) [31], the BBBike data does not show strong morning and afternoon peaks. In contrast, BBBike requests start later in the day and the number per hour remains similar during the daytime. Regular journeys like work, education or childcare require no repeated routing, so such trips are underrepresented in the data but account for a large proportion of the volume of cycle traffic.

With regard to the distribution over the course of the week, on average there are about 15 % less requests on the weekend compared with weekdays. In traffic counting data, this decrease in cycling on weekends is much greater and shows a difference of 40.3% over all counting stations in the whole year 2017 [32].

**Fig. 3:**
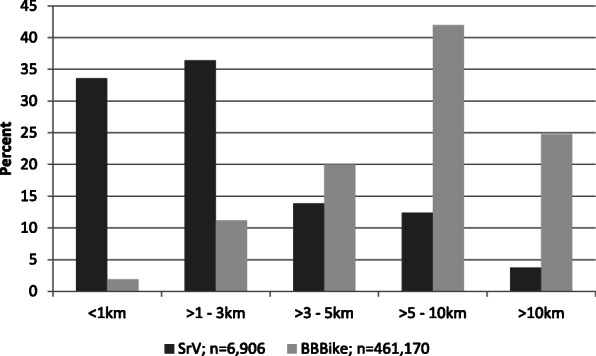
Distances of BBBike requests compared with the municipal survey. The values describe the percentage of each length category on all trips in the respective dataset

**Fig. 4:**
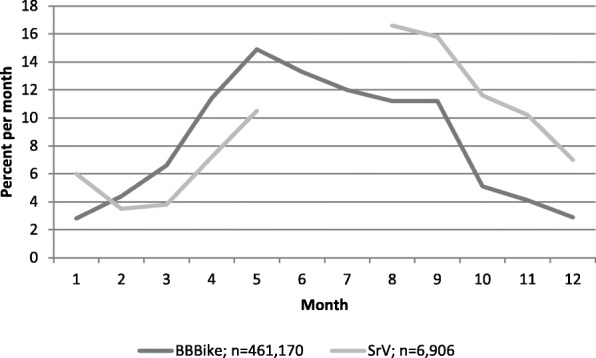
Distribution of BBBike requests over the course of the year compared with SrV. In SrV, trip data are not collected during summer holidays

In addition, the distances in BBBike requests are much longer than those of all cycle journeys reported in the municipal survey (see Fig. 3). The mean distance for BBBike requests (7.9 km) is more than twice that for journeys reported in SrV data (3.3 km). This difference in distribution indicates that the tool is being used for longer and possibly unknown routes where routing is helpful. With regard to the spatial distribution and time of year, the requests in BBBike and trips in the representative municipal household travel survey data are distributed similarly (see Fig. 4). The bike-routing engine is not designed for specific use cases like fitness cycling but for everyday traffic. Consequently, it is neither being used by a specific user group, nor does it include any gamification elements distorting the results. Overall, an extensive cross-sectional dataset is gathered over a long period of time. These data do not rely on any artificial situation that might potentially affect the individual. The enormous number of cases and the data collection, unnoticed by the user, are the primary advantages of the data. Nevertheless, the data has two main limitations: first, there is no information about the individual user who is performing the request. Second, as users are not tracked, there is no information as to whether the requesting user took the suggested route or even made the trip at all. To some extent, users search for the ideal route by making more than one request with different settings for the origin-destination relation within 5 minutes. This relates to 10,662 requests. The service should therefore be seen as an information tool. Given the high number of requests, the data reveals interesting insights into cyclists’ routing preferences. It therefore opens up the opportunity for an innovative approach which aims to investigate route search behavior.

Analysis of the routing preferences required data preparation which aimed to build a geodatabase from the log files provided by BBBike. The large volume of data was processed using Python scripts and PostgreSQL queries. This meant re-projecting the data, which initially referred to an internal coordinate system of BBBike, to the destination system WGS84. Using these coordinates, a geometry was assigned to each start and end point and visualized in QGIS.

### Cluster analysis

**Fig. 5:**
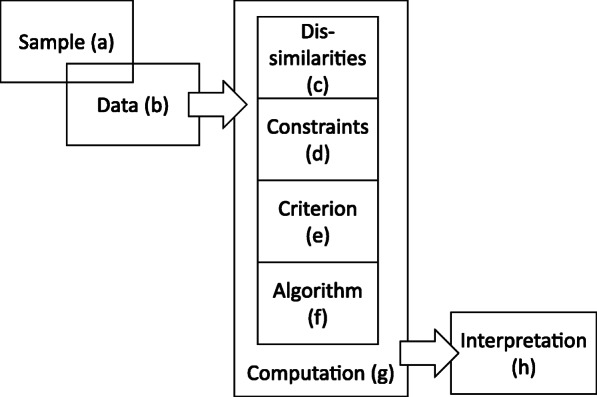
Process of the cluster analysis

The bike-routing engine allows for several different specifications of desired route characteristics in different categories. This results in a wide range of possible combinations. A hierarchical cluster analysis is performed to condense these into characteristic preference types. A hierarchical cluster analysis divides data into clusters that are as different from each other as possible and merges similar cases together into one cluster [33]. The goal is to determine a solution which on the one hand consists of as few clusters as possible, and on the other hand represents the structure of the data without losing information. The cluster analysis is structured into eight steps as shown by [34]. The process of the cluster analysis is illustrated in Fig. 5.

- **Sample (a):** The dataset described in 2.1 is used as the sample for clustering.
- **Data (b):** The characteristics of the entities on which the clustering is based are the preferences for various route attributes. These routing preferences are present as nominal data indicating preferences for various road types, surface quality and green pathways. These data include ordinal information as *no*, *weak* and *strong* preference are stated for each street type as well as for surface quality and greenery. To make this information usable, the preference settings are transformed into five ordinal variables defining the desired usage of side roads, main roads, main roads without cycle infrastructure, smooth road surfaces and green pathways with three values each. That means for all requests there is the information if no preference [0], preference [1] or strong preference [2] for each according category (residential roads, main roads, no main roads without infrastructure, avoid cobblestones, green pathways) is stated.
- **Dissimilarities (c):** The asymmetric Manhattan method as proposed by [35] is used to calculate a distance matrix for the specific case of ordinal data. In order to do so, the relative distance between every pair of observations in the dataset is calculated and organized in the distance matrix. To do this, the scale for the distance measure is treated as an interval. According to [36], the majority of authors do this so as not to lose information, even though the differences between the single values cannot be known in detail and may be different.
- **Constraints (d):** The hierarchical approach is chosen as the clustering method. In hierarchical cluster analysis, objects are merged together into clusters step by step. For each step, similarity matrices are calculated as described in (c) and objects are assigned to the cluster which fits best. Thus, the analysis produces results for a variety of cluster solutions according to the number of resulting clusters. Hierarchical clustering can thus deliver criteria to specify the optimum number of cases, while partitioning algorithms need the number of groups as input a priori. With regard to constraints, there is no need for normalization as the range and relations are identical for all variables integrated in the clustering.
- **Criterion (e):** Various measures of homogeneity exist for different types of data and approaches. By evaluating such measures, it is possible to determine the optimal number of cases in the process. The Calinski-Harabasz criterion (CHC) is used [37]. The CHC combines two important measures for evaluating each cluster solution. The total within-cluster covariance shows how compact each cluster is. A low value is preferred. The between-cluster covariance defines how different the clusters are from each other. The Calinski-Harabasz criterion is defined as

$$ {VRC}_k=\frac{S{S}_B}{S{S}_w}\times \frac{\left(N-k\right)}{\left(k-1\right)}, $$

where *SS*_*B*_ is the overall between-cluster variance, *SS*_*W*_ is the overall within-cluster variance, *k* is the number of clusters, and *N* is the number of observations.

**Fig. 6:**
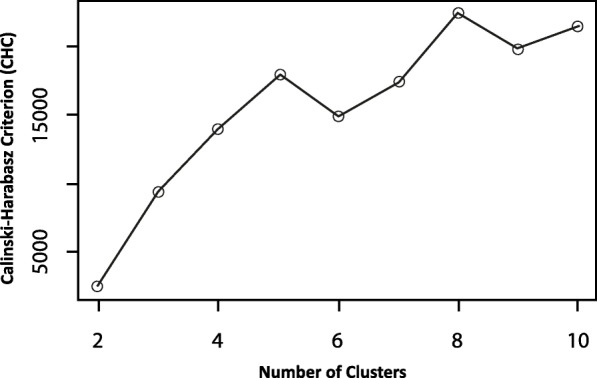
Calinsky-Harabasz criterion for different number of clusters in the data

**Fig. 7:**
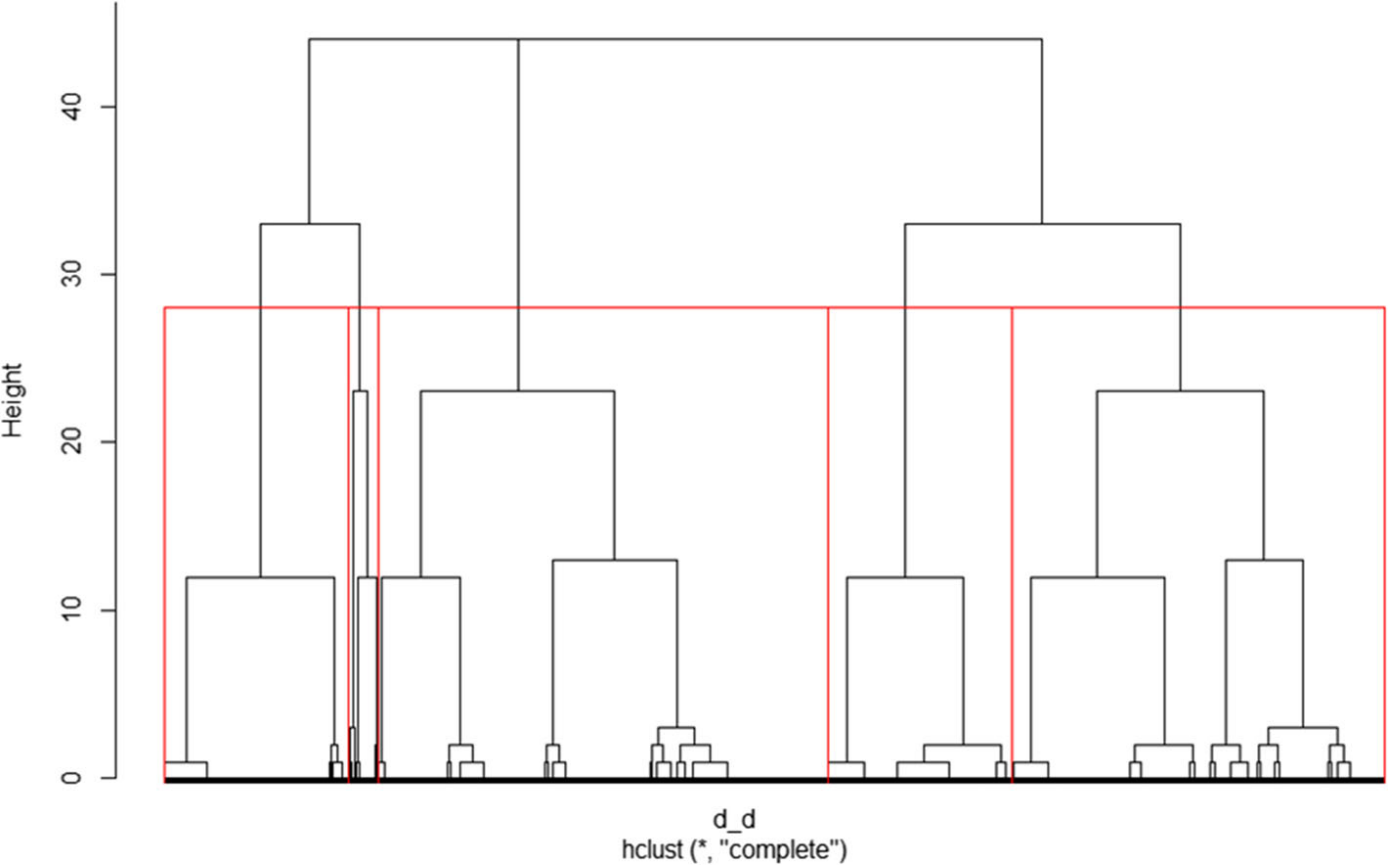
Dendrogram of the hierarchic cluster analysis

- **Algorithm (f):** The complete-linkage method is applied as the cluster algorithm to identify similar clusters. Complete-linkage measures the farthest pair of points to calculate similarity. As agglomerative hierarchical clustering, the algorithm starts from each element representing one cluster. The clusters are successively merged together until all elements are united in one cluster. This approach allows the dendrogram to be interpreted as graphical output of the clustering process (see Fig. 7). The dendrogram illustrates the tree of cluster solutions produces by the algorithm. The algorithm is relatively robust against chaining and builds rather compact clusters.
- **Computation (g):** The algorithm (f) is applied to the distance matrix (c).
- **Interpretation (h):** Interpretation and choosing the number of clusters that fits best is based on two separate evaluations. First, the CHC as described in (e) is evaluated. For the combined CHC, the best cluster solution has the highest value [37]. The CHC offers solutions with five or eight clusters (see Fig. 6).

Second, the dendrogram (Fig. 7) is evaluated. Complete-linkage allows the dendrogram to be used in graphical interpretation to choose the number of clusters that fits best (see Fig. 7). The dendrogram works as a tree diagram and displays the clustering in accordance with the sequence of the process (in which step clusters are merged together) and the distance where merging occurs (indicated by the height as shown in the Y-axis of the dendrogram). Taking the steps of merging and the high of standardized distance (Y-axis in the dendrogram) into account, a solution of five or eight clusters would be possible based on the dendrogram.

To achieve the goal of preferably few clusters, we decided for a cluster solution with five clusters to represent the data. This clustering result is indicated by red squares around each cluster in Fig. 7. The height displays the relative distance between the merged clusters in the process. It refers to the value of the according distance matrix. Interpretation of the chosen cluster solution in respect of content is described in the results section.

## Results

Now we present the results for routing preferences. First, we present a descriptive overview. Then, we carry out data processing and apply filters before applying the methodology. Groups and subsets are analyzed over time. We draw comparisons with the cycle traffic in Berlin using the official municipal household travel survey data of SrV 2013 [31]. Subsequently, we analyze preferences and present the results of the cluster analysis to describe preference types and related route characteristics.

### Overview

**Fig. 8:**
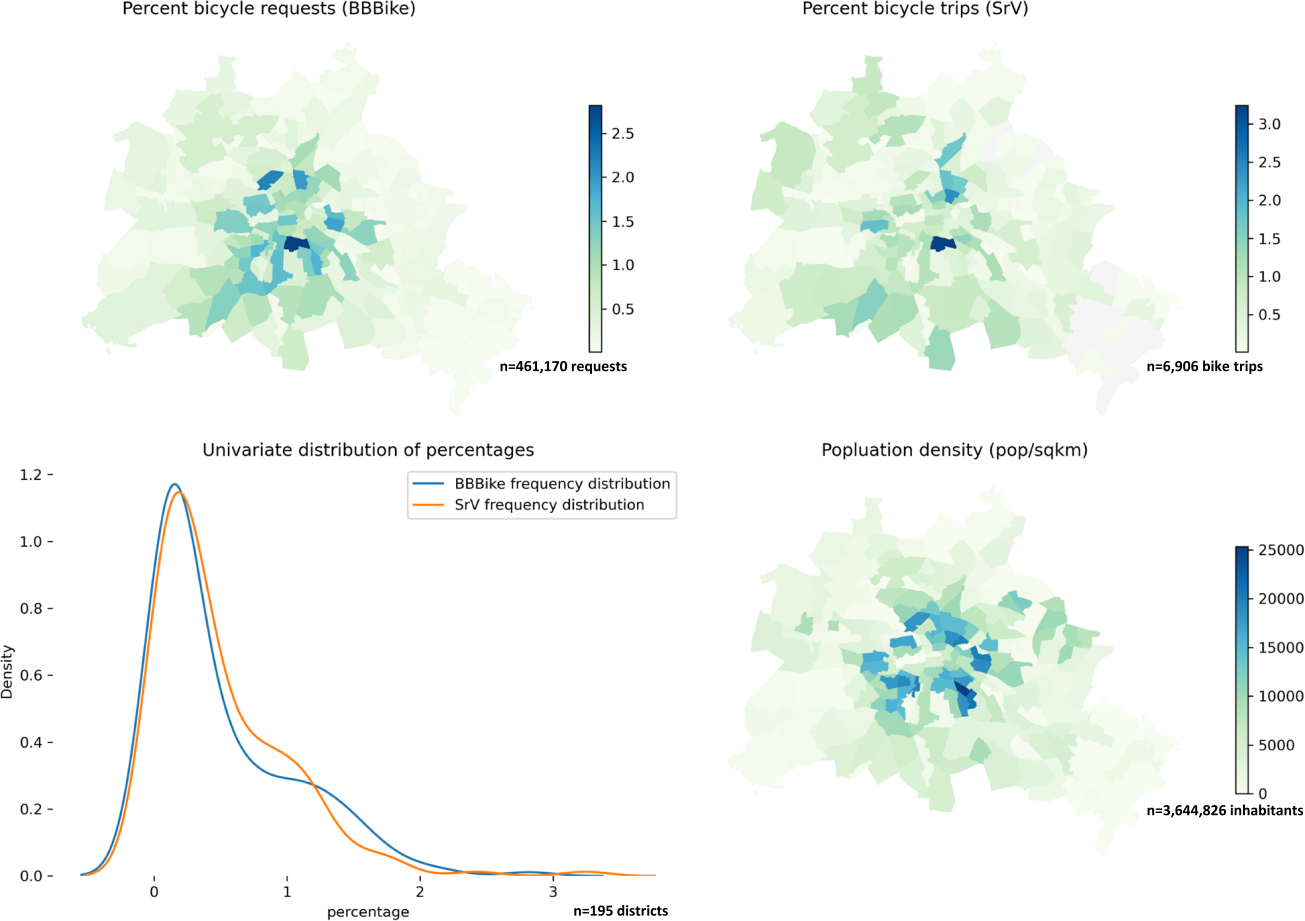
Spatial distribution of the proportion of BBBike requests in a certain district in regard to all requests (top left), the proportion of SrV trips starting in a certain district in regard to all trips (top right),), frequency distribution of percentages of BBBike and SrV across all districts (bottom left) and population density (bottom right)

The mean distance in BBBike requests shows a strong peak during summer. With regard to the spatial distribution of requests in terms of start and destination locations, we can observe a concentration in the inner city. The heatmap of destination locations does not differ substantially from this picture. Figure 8 compares the spatial distribution of starting locations of BBBike requests to the starting locations of cycle trips according to SrV and the population density in Berlin. In addition, we compare the frequency distributions of BBBike and SrV. As seen in Fig. 8, these distributions appear similar in general. As assumed, the starting locations of both datasets, BBBike and SrV peak in the inner city. Both distributions also show more trips in western outskirts than in eastern districts. More precisely, BBBike routing requests seem to interrelate stronger to population density than the bike trips in SrV data do. Hence, BBBike requests clearly peak in dense inner-city districts. The frequency distributions of BBBike and SrV are very similar.

**Fig. 9:**
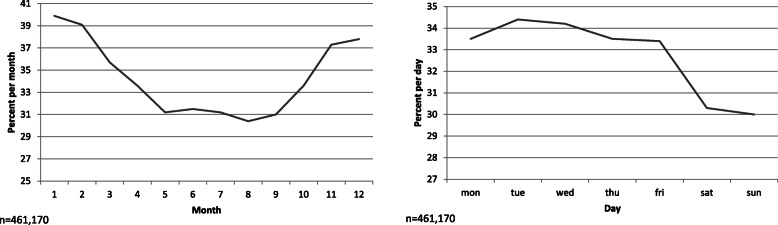
Share of default requests in all requests over the course of the year (left) and the course of the week (right)

To gain insights into routing preferences, we analyzed BBBike-routing preferences as seen in Table 1. When examining the data, we see a high proportion of default requests. The request is defined as default when only the origin and destination are given but no routing preference is stated in any of the settings. These default requests make up 36.1% or 166,341 observations. It can clearly be seen that much lower rates of default requests occur on weekends than weekdays and in summer over winter as shown in Fig. 9. Accordingly, trips using the default settings have a shorter distance than those with indicated preferences (6877.3 vs. 7487.7 on average). BBBike gives the shortest route whenever no preference is specified.

The distribution of other preference settings differs over the course of the year. The preference for main roads makes up only 2 % in May and reaches the maximum of 4.3% of all requests in January.

**Fig. 10:**
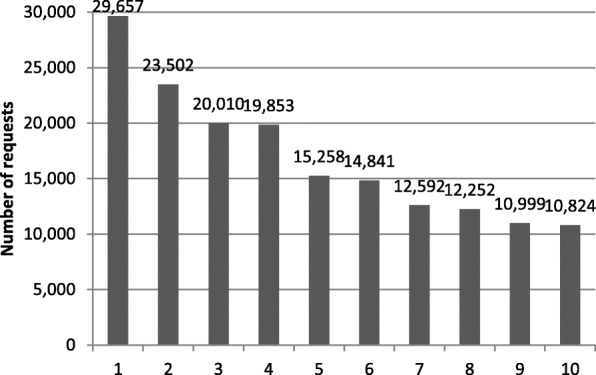
Top ten most common preference settings (without default queries): 1: Avoid cobblestones and bad surfaces. 2: Prefer residential roads, prefer green pathways, avoid cobblestones and bad surfaces. 3: Prefer green pathways. 4: Prefer residential roads, avoid cobblestones and bad surfaces. 5: Prefer residential roads. 6: Prefer residential roads, prefer green pathways. 7: Avoid main roads without cycle paths, avoid cobblestones and bad surfaces. 8: Avoid main roads without cycle paths, prefer green pathways, avoid cobblestones and bad surfaces. 9: Avoid main roads without cycle paths/bus lanes, avoid cobblestones and bad surfaces. 10: Prefer green pathways, avoid cobblestones and bad surfaces

Figure 10 summarizes the ten most frequently used combinations of settings for individual requests. The default requests, clearly dominating the individual settings, are not displayed. The six most common requests either include the preferred use of side roads or display a simple preference for either green pathways or smooth surfaces. Accordingly, when any routing preference is specified by the user (no default queries), the most common individual preference is related to surface quality (29,657 requests).

**Fig. 11:**
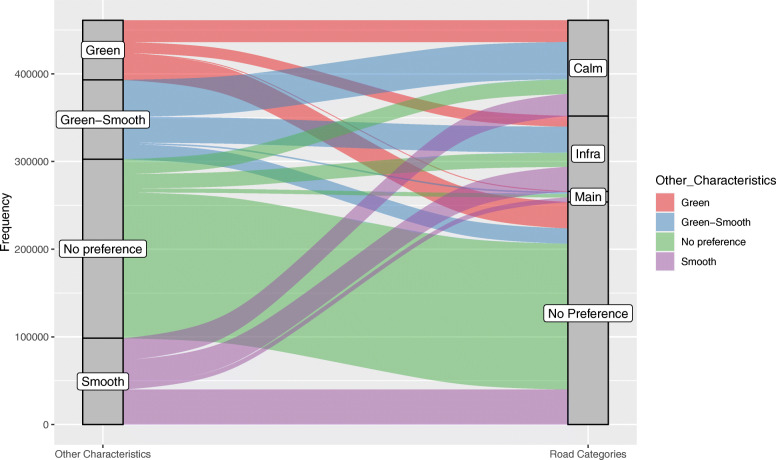
Interrelations between preferences for other characteristics (left column) and road categories (right column). Other characteristics: prefer green pathways (Green), avoid bad surfaces (Smooth), prefer green pathways and avoid bad surfaces (Green-Smooth); Road preferences: prefer calm residential roads (Calm), avoid main roads without cycle infrastructure (Infra), prefer main roads (Main); simple and strong preference summed up

Figure 11 illustrates the interrelations between preferences for road categories and preferences for other characteristics like greenery or surface quality. In the flow diagram the size of the bars indicate the overlap between the characteristics on left side of the diagram and the road categories on the right side of the diagram. It therefore illustrates the structure of the data. We can grasp the importance of the interrelations between specific characteristics from this. For example, a preference for green pathways is often stated solely (lowest red bar linking “Green” to “No Preference”) or together with a preference for calm side roads (top red bar). On the contrary, the joint preference for green pathways and smooth surface mainly goes together with a preference for calm side roads (top blue bar) and a preference for infrastructure (second blue bar linking “Green-Smooth” to “Infra”). A preference for main roads is mainly linked to no additional preference (third green bar) and preference for smooth surfaces (third purple bar) while interrelations to any setting preferring greenery are limited (red and blue bars). As obvious, by far the largest interaction is shown by the default queries with combine no stated routing preference for both, road categories and other characteristics (vast green bar). For a detailed list of all intersected preferences including the exact quantification see Table 3 in the appendix.

Comparing preference settings between city regions, it is seen that green pathways and calm roads are requested less in the inner city than in outer parts of the city. More precisely, requests preferring green pathways in the center account for nine percentage points less than in outer parts. The difference with regard to calm roads in the inner city is three percentage points. In contrast, requests searching for the shortest route occur more often. Other preferences do not show noticeable differences.

For a detailed overview of the length and overlap of different routes for varying settings in different urban contexts, see Table 4 in appendix and figures in the appendix. These provide an impression of the sensitivity of the routing algorithm and the different routes provided for different routing preferences.

### Results of the cluster analysis: preference types

**Fig. 12:**
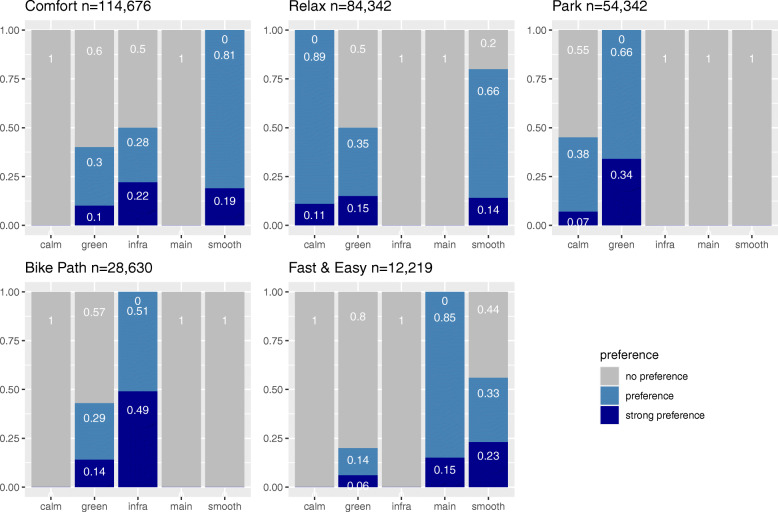
Percentages of preferences in resulting clusters

The cluster analysis as described in 2.2 results in five clusters. These clusters characterize the preferences observed in the requests and may be described as content-related. Figure 12 gives an overview about the distribution of routing preferences in each cluster and shows the number of requests per cluster.

In the cluster **Comfort,** avoiding disturbances caused by bad surface quality is determinant. All requests wish to avoid bad surfaces with 81% aiming to avoid cobblestones and bad surfaces and 19% wishing to use only very good surfaces. In addition, 50% of the requests wish to avoid main roads without cycle path (or bus lanes). Green pathways are less relevant than on average with 30% preferring and 10% strongly preferring them.

The clusters **Relax** and **Park** show preferences for combining calm side roads and green pathways. In the cluster **Relax,** the request for side roads dominates. Also, in **Relax,** a preference for smooth surfaces is given in 80% of the requests, while in **Park** smooth surface is not requested at all. In **Park** the preference for green pathways is seen in all requests.

The cluster **Bike Path** shows a low preference for green pathways, while all requests wish to avoid main roads without cycle path (or bus lanes). Here, smooth surface is not requested at all.

By far the smallest cluster **Fast & Easy** shows a preference for cycling along main roads regardless of the existence of cycle infrastructure. The importance of green pathways is the lowest of all clusters. The preference for smooth surfaces is above average.

In addition to the results of the cluster analysis, the **Default** queries represent the largest group. Here, the users did not state any routing preference and did confirm the default setting of the bike-routing engine. According to BBBike, in these requests the shortest bike route is provided. The detailed characteristics of each cluster are shown in Fig. 12. The figure shows the distribution of preference settings, which the users of the bike-routing engine chose in each cluster (see Table 1). This summarizes the main results of the cluster analysis.

Three major results are particularly interesting: first, a preference for green pathways is seen in all clusters to some extent. Second, smooth surface plays a major role in three clusters. Third, two clusters combine preferences for greenery and calm side roads.

## Discussion

This study analyzes the detailed requests of a bike-routing engine. In contrast to conventional methodologies like SP or RP studies, a non-personalized big data basis has been clustered in order to generate routing preference types that make it possible to infer the importance of road characteristics to cyclists from a user’s perspective. The outcomes show stable clustering results and clear preferences towards certain infrastructural facilities (see section 3). Earlier research found that analyzing recorded data on search behavior may generally be used to estimate consumer preferences [38]. The present approach has several advantages. Due to the efficient way that data is collected, it is possible to gather a very large dataset of almost half a million cases over a long observation period of 1 year. It therefore becomes possible to use a full sample of the requests in the bike-routing engine without being potentially distorted by a survey situation. This eliminates several disturbing influence factors like the social desirability bias, the observer bias or the non-response bias. In addition, we do not rely on a conceptual choice experiment or the normative generation of alternatives. As shown in the literature section, previous research does not always come up with clear and consistent results. Against this background, this approach provides findings from a different point of view, which help to assess the integrated overall view of route choice behavior in the context of validating earlier results. These main issues are presented below.

At around one third, a very large proportion of requests were executed in default mode. To some extent the large number of default requests might be explained by users who do not read the explanation and do not change the default setting due to a lack of attention. The settings used for the request are displayed in drop-down menus after typing origin and destination and have to be confirmed before the route is calculated. When used on purpose, these requests represent a preference for the shortest route. Searching for the shortest route is more important in the winter months than in summer and on weekdays compared with weekends. As described, it is assumed that the requests pertain largely to leisure and sporadic trips. Earlier research found that on repeated and especially on non-leisure trips, cyclists tended to choose the shortest route more often than on routes for other purposes [7]. Accordingly, disutility of the absence of bike infrastructure appears lower on commuting trips [8]. This suggests that the preference for the shortest route may even be stronger than the sample reveals. It should be clarified that this is especially true for male frequent cyclists [7]. In this matter, researching route preferences mainly on leisure trips may reveal more information on desired route characteristics, as time constraints are less important and influence route choices to a lesser extent. It is assumed that when differentiating route choice behavior between trips with different purposes, the pivotal factor is time pressure rather than differences in desired route characteristics.

If any routing preference is specified, the most important setting (more than half of all requests) is to avoid bad surfaces. Avoiding bad surfaces is the most frequently used individual setting and accompanies all preferred road categories. A slightly lower preference on main roads might be explained by the fact that there are few main roads with bad surface quality in Berlin. The results therefore clearly demonstrate the dominant significance of smooth surfaces. This has to be seen within the context of the investigation area in Berlin. As explained, a number of residential streets, are paved with cobblestones and have bad surfaces. Consequentially, the fact that participants in Berlin are highly aware of surface quality and the existence of methodological differences may explain the discrepancy in respect of earlier stated preference studies which concluded that surface quality had limited importance compared with other factors [13, 16].

Compared to earlier results, in this study more requests show a preference for cycling in mixed traffic on calm roads over separated facilities [8, 11, 12, 14–16]. With a difference of five percentage points, prioritizing calm roads is more common than accepting routes which include segments on main roads with cycle infrastructure. The latter are classified in the cluster *Bike Path* and partly in *Comfort*. This discrepancy may be partially explained by well-designed images of cycle infrastructures in stated preference studies compared with a rather more moderate design and condition of such infrastructures in Berlin since large parts of the bike infrastructure originate from the 80th when different design standards were applicable.

In terms of the relevance of off-street cycling facilities, i.e. green pathways, the results are in line with several earlier studies revealing their strong effect [8, 14, 16, 39].

On the whole, the cluster analysis shows that specific combinations of different preference settings are more common than others. For example, calm roads are often used together with a preference for green and/or smooth routes as seen in the clusters *Relax* and *Park*, while main roads are combined with smooth surfaces but very rarely with green routes. The cluster solution identifies the interrelation by condensing 63 possible settings into five preference types plus the default cluster which probably presents a preference for the shortest route. The clustering shows a stable solution and represents combinations of preferences with clear priorities in each cluster. Accordingly, characteristic desires can be condensed into just a few combinations of settings. These individual preference types are reflected by the clusters *Relax* and *Park* combining calm roads with green pathways, *Comfort* and *Bike Path* looking for smooth surfaces and (partly) avoiding main roads without cycle infrastructure, *Fast & Easy* desiring main roads regardless of cycle infrastructure and *Short* with the search for the shortest route using the default settings. These clusters show preference settings which differ strongly from each other, illustrating that there is no ideal route and no ‘one-size-fits-all-approach’, but rather distinct individual and trip-related preferences that determine route choices.

As described, Berlin is seen as a suitable case study. Given the nearly half a million observations recorded and the long history of the bike-routing engine in Berlin, it may capture a sufficient picture of bicycle transport in Berlin considering the limitations described below. More recently, the bike-routing engine has become available in many other cities which will make it possible to verify to what extent resulting clusters can be generalized.

The limitations of the present study are discussed below. Individual users are unknown due to the methodological approach and the way the data is recorded. So, unlike previous studies, we cannot evaluate the routing preferences in groups based, for example, on sociodemographic features or level of cycle confidence [8, 11, 16, 40]. The participants of this study, and accordingly the results, cannot be regarded as representative of the municipal population but should reflect cyclists in the investigation area with an affinity to ICT. Most importantly, we are researching the people who already cycle and the conclusions drawn can only be based on them. Given the methodological approach, we can only research the users of the bike-routing engine. In addition, other than modelling approaches (RP or SP) we cannot quantify trade-off e.g. between travel times and route characteristics.

As we do not know the individuals behind the requests, the resulting preference types pertain to trips rather than to individuals. Accordingly, it is possible that an individual user shows different routing preferences for different occasions. Also, as the bike-routing engine provides routes but does not track cyclists, it is not known to what extent using the tool actually results in traveling the proposed route. Most precisely, the data reflects route searching or planning behavior rather than route choice behavior. Nevertheless, earlier research justifies the main idea of the approach [38]. As described, in a limited amount of cases users even try different settings for the same origin-destination relation. With regard to the temporal aspect, we cannot prove for certain whether requests for a specific trip are made immediately before this trip. However, the distribution of the requests over the course of the day, week and year appears plausible. If we assume a close time connection between request and planned starting time of the trip, these distributions indicate primary but not sole use for leisure and sporadic trips. In addition, the tool’s purpose for navigation beyond known routes or a well-known neighborhood narrows the representative nature of the data. It is obvious that no repeated navigation is needed for commuting trips or short trips in the neighborhood. Thus, the navigation is used for much longer trips than the mean distance for cycling trips according to the municipal data (SrV). As a result, there is less information on journeys cycled on a regular basis as well as short trips which do, however, account for large proportions of the road traffic.

Finally, the analysis carried out in this paper can only research preferences based on choosing alternatives from the predefined options the tool provides. Any further preferences remain hidden. For example, all types of infrastructure along main roads like bike lane, bike path or protected bike lane create one category. The type of bicycle infrastructure is not differentiated in the data. In that context, the interpretation of the default settings matters. According to BBBike, in the default setting the shortest bike-routing is computed. From a user’s perspective, this is understandable since every additional preference specified potentially leads to longer trips. The observation of significantly less default queries both in summer and on weekends compared to winter and weekdays suggests the interpretation of using default for the shortest path since the share of these queries declines when time constraints and weather conditions are likely to allow for longer bike trips or more precisely, detours are more acceptable. Hence, it is likely that the majority of the default queries pertain to a preference for the shortest route. The importance of short trips is plausible as travel time is major impacting variable in transport research [41, 42]. Nevertheless, it is also possible that parts of the users do not pay attention to the possible routing settings and confirm the default setting displayed be the tool for no specific reason. This has to be kept in mind when interpreting the concluded desire for shortest trips.

## Conclusion

The present study provides insights into cyclists’ route preferences by analyzing an extensive dataset of requests with according routing preference settings collected in the bike-routing engine BBBike. Compared with previous studies, this study uses a different type of data and a different approach. The findings gathered under different circumstances show several relevant findings. It is seen that diverse routing preferences can be condensed into six trip-related preference types which differ strongly from each other. Thereby, the largest of these routing preference types is defined by the default settings of the routing engine and therefore searches for the shortest route without limitations on route specifics. Compared to prior findings, in the present study with the according setting, surface quality and using side roads appear to be more important than separate cycle infrastructure along main roads. Apart from that, for a small proportion of trips, cyclists prefer main roads irrespective of cycle infrastructure.

When providing recommendations for designing a bike-friendly city, the following key messages become apparent: First, given the dominant preference for the shortest route as indicated by the default queries, there is a strong need for short cycle connections through the city. On one hand, this strengthens the potential for cycle super-highways or express routes for cyclists as these enable fast transit. On the other hand, it shows a need for a dense network ensuring direct cycle connections through the city. Second, providing a well signposted coherent network of cycle connections on calm side roads combined with well-maintained surface quality appears to be a key point. A strategy such as this satisfies a greater demand than providing a network of separated cycle facilities along main roads. Given the opposing preferences, an integrated strategy should take both into account. Third, when planning cycle routes, specific preference types need to be considered to consistently meet the demand. For example, combining segments on calm side roads with segments through parks fulfills connected preferences, while combining cycle facilities on main roads with green segments does not.

## Figures and Tables

**Fig. 13:**
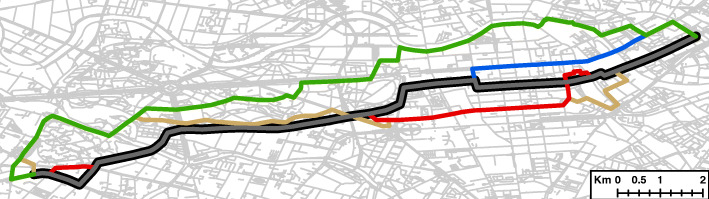
Inner city routes

**Fig. 14:**
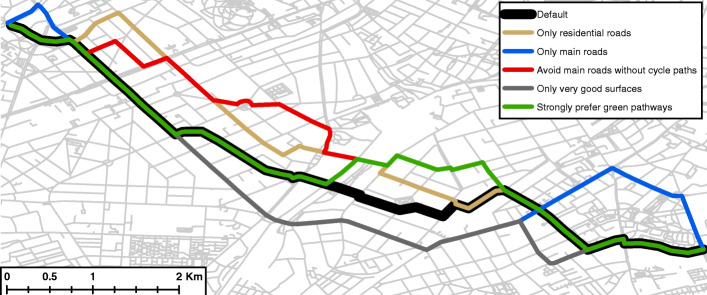
Edge of town routes

**Fig. 15:**
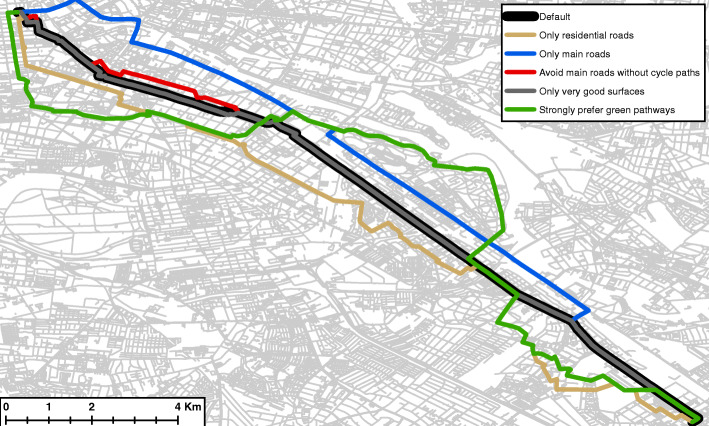
Edge of town to city center routes

## Data Availability

The data that support the findings of this study are available upon reasonable request from the authors.

## Appendix

**Table 2:** Overview of cited studies

Author	Year	Variables used in the model	Type of analysis	Country	Investigation area	Local cycle mode share
Bernardi, La Paix Puello, Geurs	2018	Link type [type of road or bike infrastructure], Link quality, Link beauty, Link traffic nuisance	Revealed preference	Nether-lands	Countrywide	Not stated
Ghanayim, Bekhor	2018	Total route length, Route length on streets with bike paths, Route length on urban arterials and highways, Average street length, Dwelling units / m, Route length “near sea”, Route length “near park”	Revealed preference	Israel	Tel Aviv metropolitan area	1,6%
Prato, Halldórsdóttir, Nielsen	2018	Distance, Wrong way, Left turns, Right turns, Bicycle infrastructure type, Bicycle facility type, Cumulative elevation gain, Surface type, Number of intersections, Motorized road type, Motorized free speed, Number of motorized traffic lanes, Land-use designations	Revealed preference	Denmark	Copenhagen	37% commuting trips
Vedel, Jacobsen, Skov-Petersen	2017	Road environment, Cycle track, Green surroundings, Crowding (other cyclists on the route), Stops (on the route), Route length	Stated preference	Denmark	Copenhagen	35% of all trips
Aldred, Elliott, Woodcock, Goodman	2016	Varying	Review on stated preference surveys	Varying	Varying	Varying
Buehler, Dill	2016	Varying	Review	World	Varying	Varying
Mertens, Van Dyck, Ghekiere, De Bourdeaudhuij, Deforche, Van de Weghe, Van Cauwenberg	2016	Type of cycle path, speed limit, speed bump, vegetation, evenness of the cycle path surface, general upkeep, traffic density	Stated preference	Belgium	Flanders	14% of trips shorter than 5 km
Winters, Teschke, Brauer, Fuller	2016	Bike Score (10-unit change), Destinations/Connectivity Score, (10-unit change), Bike Lane Score (10-unit change), Hill Score (10-unit change), Bike Score (categorical), City	Cross-sectional	US/ Canada	24 cities	1,9% (mean) commuting trips
Nielsen, Olafsson, Carstensen, Skov-Petersen	2013	Distance to retail concentration, Train station within 1 km, Population density within 1.5 km, Public transport departures within 500 m, Retail jobs/resident within 500 m, Topology as elevation range within 1.5 km, Intersection pr. Network dist. Within 1.5 km, Intersection pr. Network dist. Within 500 m (Ln), Residence is a flat, Copenhagen or Frederiksberg (Place dummy), City of Odense (Place dummy), Respondent has driver’s license, Occupation: student, Occupation: full time employment, Education: medium, Education: long (academic), Family type: single, Household income/adult, Personal income, Gender	Cross-sectional	Denmark	Denmark	23% Individuals who cycle
Broach, Dill, Gliebe,	2012	Bridge with on-street bike lane, Proportion of route along links with [varying] upslope, Distance of route, Path size, Left turn without traffic signal and parallel [varying] traffic volume, Proportion of route on designated bicycle boulevard, Proportion of route on off-street, regional bike path, Proportion of route on streets with [varying] traffic volume without a bike lane, Left turns and straight movements through traffic signals per mile, Turns or straight movements through stop signs per mile, Left and right turns per mile, Right turns at unsignalized intersections with cross traffic volume 10,000+ per day, Left turns and through movements at unsignalized intersections with [varying] cross traffic volume	Revealed preference	USA	Portland	Not stated
Caulfield, Brick, McCarthy	2012	Adjacent traffic speed (km/h), Type of infrastructure, Travel time (min), Number of junctions on route, Cycle traffic on route	Stated preference	Ireland	Dublin	Not stated
Buehler, Pucher	2011	Bike share of commuters, Bike commuters per capita, Bike lane supply, Bike path supply, Cycling safety, College students, Car access, Sprawl index, Public transport supply, Gasoline price, Hot weather, Cold weather, Annual precipitation	Cross-sectional	USA	90 cities	0,8% (mean) Commuting trips
Winters, Teschke	2010	Major streets, residential streets, rural roads and highways, off-street paths, cycle paths next to major roads but physically separated from traffic, road markings, bicycle lanes, traffic calming, route surfaces, car parking	Stated preference	Canada	Vancouver metropolitan area	~ 2%
Winters, Teschke, Grant, Setton, Brauer	2010	Gross population density, % of land area with green cover, average air pollution (ppb NO2), variation in elevation, % of road segments > 10% slope, traffic calming features, stencils, bike route signs, traffic crossings with bike activated signals, ratio of 4 way intersections: all intersections, *% of land area with use: (*agriculture, commercial, education, entertainment, industrial, office, park, single family residence, multifamily residence, land use mix)	Revealed preference (shortest)	Canada	Vancouver metropolitan area	1,7% for work trips
Garrard, Rose, Lo	2008	Type of bicycle facilities (path, lane, no)	Observing	Australia	Melbourne	1,2%
Hunt, Abraham	2007	Availability of showers at destination, Availability of secure parking at destination, Minutes riding on roadways in mixed traffic, Minutes riding on designated bike lanes on roadways, Minutes riding on bike paths shared with pedestrians	Stated preference	Canada	Edmonton	Not stated
Moudon, Lee, Cheadle, Collier, Johnson, Schmid, Weather	2005	Age in years, Gender, Race, Marital status, General health, Income, Own a bicycle?, Number of cars in household, Vehicle miles traveled per month, Exercise at home?, Use transit?, Work hours per week, Vigorous activity, Number of facilitators of cycling mentioned, Total household location factors -Proximity to recreational destinations, Perceived presence of, Benefits of physical activity, Presence of amenities for cycling and jogging in the neighborhood, High social support for walking and cycling in the neighborhood, High visual quality of the neighborhood, Presence of destinations in neighborhood, Presence of auto-oriented facilities in the neighborhood, Problems related to automobiles in neighborhood, Percentage of streets lined with bicycle lanes, Distance to the closest trail, Number of parks within the 3 km buffer, Number of destinations within the closest NC6 (sports facility and school), Size of the closest NC3 (grocery and restaurant), Area of convenience stores within the 3 km buffer, Number of parcels within the closest NC10 (office, fast food, and clinic/hospital)	Cross-sectional	USA	King County, Washington	< 1%
Stinson, Bhat	2003	Roadway class, Parallel parking permitted, Bicycle facility type, Bridge type, Hilliness, Riding surface, Travel time, Facility continuity, Number of stop signs per mile, Number of red lights, Number of major cross-streets	Stated preference	USA / Canada	Countrywide	varying
Abraham, McMillan, Brownlee, Hunt	2002	Total cycling time including stops at red lights and stop signs, time on arterial roads, time on arterial roads with wide curb lane, time on arterial roads with bicycle lane, time on residential roads, time on bike route consisting of residential roads, time on bicycle pathways alongside arterial road, time on bicycle pathways in park area, Parking facility available at destination, Cost for parking facility, Other facilities available at destination, Cost for other facilities	Stated preference	Canada	Calgary	Not stated
Aultmann-Hall	1996	Turns, Turns per km, Signals, Signals per km, Major signals, Proportion of movements between a major and minor road with a signal, Proportion of movements from a minor/path to a minor/path with a signal, Proportion of route on arterial roads, Proportion of route on collector roads, Proportion of route on local roads, Proportion of route off-road, Road bridges, Travel on grade (km), Level railway crossings	Revealed preference (shortest)	Canada	Guelph, Toronto, Ottawa	Not stated

**Table 3:** Distribution of preferences for road categories (x-axis) intersected with preferences for other characteristics (y-axis)

		Calm	Infra	Main	No Preference	Overall
Green	n	24,887	12,260	876	30,075	**68,098**
	% in column	22.8%	14.3%	7.2%	11.9%	14.8%
	% in row	36.5%	18.0%	1.3%	44.2%	100.0%
	% in total	5.4%	2.7%	0.2%	6.5%	14.8%
Smooth	n	25,161	27,993	5317	39,951	**98,422**
	% in column	23.0%	32.6%	43.5%	15.7%	21.3%
	% in row	25.6%	28.4%	5.4%	40.6%	100.0%
	% in total	5.5%	6.1%	1.2%	8.7%	21.3%
Green- Smooth	n	42,164	29,347	1547	17,385	**90,443**
	% in column	38.6%	34.1%	12.7%	6.9%	19.6%
	% in row	46.6%	32.4%	1.7%	19.2%	100.0%
	% in total	9.1%	6.4%	0.3%	3.8%	19.6%
No preference	n	17,017	16,370	4479	166,341	**204,207**
	% in column	15.6%	19.0%	36.7%	65.6%	44.3%
	% in row	8.3%	8.0%	2.2%	81.5%	100.0%
	% in total	3.7%	3.5%	1.0%	36.1%	44.3%
Overall	**n**	**109,229**	**85,970**	**12,219**	**253,752**	**461,170**
	% in column	100.0%	100.0%	100.0%	100.0%	100.0%
	% in row	23.7%	18.6%	2.6%	55.0%	100.0%
	% in total	23.7%	18.6%	2.6%	55.0%	100.0%

Road preferences: prefer calm residential roads (Calm), avoid main roads without cycle infrastructure (Infra), prefer main roads (Main); other characteristics: prefer green pathways (Green), avoid bad surfaces (Smooth), prefer green pathways and avoid bad surfaces (Green-Smooth); simple and strong preference summed up. **Reading example:** the first cell shows that 24,887 requests combine a preference for calm roads (column) with that for green pathways (row). The percentage values show that 22.8% of requests which give a preference for calm roads (column) also prefer green pathways (row). At the same time, in 36.5% of requests preferring green pathways, there is also a preference for calm roads. In total, the combination preferring calm roads and green pathways makes up 5.4% of all requests

**Table 4:** Comparison of length and overlap for exemplary routes

Relation	Inner city routes [City center east/ Alexanderplatz –City center west/ Breitscheidplatz]		Edge of town routes [Freie Universität Lankwitz/ Malteserstr –Freie Universität Dahlem/ Thielplatz.]		Edge of town to city center routes [Humboldt Universität Adlershof/ Rudower Chaussee –Humboldt Universität Mitte/Unter den Linden]	
[km]	Length	Length identical with default	Length	Length identical with default	Length	Length identical with default
Default	6.25	6.25	6.57	6.57	14.31	14.31
Prefer residential roads [calm]	6.63	2.38	6.75	2.37	15.83	7.50
Use only residential roads [calm*]	7.39	0.83	6.92	2.82	16.91	0.94
Prefer main roads [main]	6.39	4.19	7.11	1.82	14.93	4.07
Use only main roads [main*]	6.39	4.19	7.44	1.33	15.19	3.09
Avoid main roads without cycle paths/bus lanes [infra]	6.46	4.14	6.83	2.53	14.53	11.35
Avoid main roads without cycle paths [infra*]	6.34	3.60	6.83	2.53	14.55	11.52
Avoid cobblestones and bad surfaces [smooth]	6.25	6.25	6.75	2.75	14.31	14.31
Use only very good surfaces [smooth*]	6.25	6.25	6.75	2.75	14.32	13.98
Prefer green pathways [green]	7.47	0.00	6.57	6.57	17.27	6.20
Strongly prefer green pathways [green*]	7.54	0.00	6.76	4.98	18.93	2.11

For a clearer overview, only strong preferences are displayed on the maps

Strong preferences are indicated with *

## Footnotes

1 For a detailed overview of all studies cited, regarding framework conditions of the area under investigation and variables included in the model see table 3 in the appendix.
